# Epidemiological Investigations of Four Cowpox Virus Outbreaks in Alpaca Herds, Germany

**DOI:** 10.3390/v9110344

**Published:** 2017-11-18

**Authors:** Almut Prkno, Donata Hoffmann, Daniela Goerigk, Matthias Kaiser, Anne Catherine Franscisca van Maanen, Kathrin Jeske, Maria Jenckel, Florian Pfaff, Thomas W. Vahlenkamp, Martin Beer, Rainer G. Ulrich, Alexander Starke, Martin Pfeffer

**Affiliations:** 1 Clinic for Ruminants and Swine, Faculty of Veterinary Medicine, University of Leipzig, An den Tierkliniken 11, 04103 Leipzig, Germany; ap81weru@studserv.uni-leipzig.de (A.P.); mkaiser@vetmed.uni-leipzig.de (M.K.); alexander.starke@vetmed.uni-leipzig.de (A.S.); 2 Institute of Diagnostic Virology, Friedrich-Loeffler-Institut, Federal Research Institute for Animal Health, Südufer 10, 17493 Greifswald-Insel Riems, Germany; Donata.Hoffmann@fli.de (D.H.); Maria.Jenckel@fli.de (M.J.); Florian.Pfaff@fli.de (F.P.); Martin.Beer@fli.de (M.B.); 3 Veterinary practice Dr. Daniela Goerigk, Naundorfer Str. 9, 04668 Schkortitz, Germany; info@tierarzt-goerigk.de; 4 Institute of Animal Hygiene and Veterinary Public Health, Centre for Veterinary Public Health, Faculty of Veterinary Medicine, University of Leipzig, An den Tierkliniken 1, 04103 Leipzig, Germany; acfvmaanen@yahoo.co.uk; 5 Institute of Novel and Emerging Infectious Diseases, Friedrich-Loeffler-Institut, Federal Research Institute for Animal Health, Südufer 10, 17493 Greifswald-Insel Riems, Germany; kathrin.jeske@fli.de (K.J.); Rainer.Ulrich@fli.de (R.G.U.); 6 Institute of Virology, Faculty of Veterinary Medicine, University of Leipzig, An den Tierkliniken 29, 04103 Leipzig, Germany; vahlenkamp@vetmed.uni-leipzig.de

**Keywords:** cowpox virus, *Orthopoxvirus*, South American camelids, common vole (*Microtus arvalis*), reservoir host, spill-over infection, zoonosis

## Abstract

Four cowpox virus (CPXV) outbreaks occurred in unrelated alpaca herds in Eastern Germany during 2012–2017. All incidents were initially noticed due to severe, generalized, and finally lethal CPXV infections, which were confirmed by testing of tissue and serum samples. As CPXV-infection has been described in South American camelids (SACs) only three times, all four herds were investigated to gain a deeper understanding of CPXV epidemiology in alpacas. The different herds were investigated twice, and various samples (serum, swab samples, and crusts of suspicious pox lesions, feces) were taken to identify additionally infected animals. Serum was used to detect CPXV-specific antibodies by performing an indirect immunofluorescence assay (iIFA); swab samples, crusts, and feces were used for detection of CPXV-specific DNA in a real-time PCR. In total, 28 out of 107 animals could be identified as affected by CPXV, by iIFA and/or PCR. Herd seroprevalence ranged from 16.1% to 81.2%. To investigate the potential source of infection, wild small mammals were trapped around all alpaca herds. In two herds, CPXV-specific antibodies were found in the local rodent population. In the third herd, CPXV could be isolated from a common vole (*Microtus arvalis*) found drowned in a water bucket used to water the alpacas. Full genome sequencing and comparison with the genome of a CPXV from an alpaca from the same herd reveal 99.997% identity, providing further evidence that the common vole is a reservoir host and infection source of CPXV. Only in the remaining fourth herd, none of the trapped rodents were found to be CPXV-infected. Rodents, as ubiquitous reservoir hosts, in combination with increasingly popular alpacas, as susceptible species, suggest an enhanced risk of future zoonotic infections.

## 1. Introduction

The Alpaca (*Vicugna pacos*) is one of four species of South American camelids (SACs). Originating from the Andean plateaus in South America, they were introduced to Europe about 40 years ago [1]. Alpacas are commonly held in flocks in open stabling or pasture feeding [1]. Primarily used as livestock for breeding for wool and meat production, they are also kept as pets for landscape conservation, animal-assisted therapy, or recreational activities like trekking [1,2,3,4]. Compared to other indigenous livestock, infectious diseases in SACs are still insufficiently studied. Nevertheless, there is a noticeable increase in research [4,5,6,7,8,9,10].

Cowpox virus (CPXV) is a member of the family *Poxviridae*, genus *Orthopoxvirus* (OPV) and endemic in Western Eurasia [11,12]. CPXV infections have been increasingly described in domestic (cats, dogs, horses) [13,14,15,16] and zoo animals (e.g., elephants, rhinoceros, okapis, lions, cheetahs, anteaters, monkeys) [11,17]. Zoonotic virus transmission to humans occurs via direct contact to infected animals [11] resulting in sporadic cases. 

Domestic and zoo animals as well as humans are suspected to be accidental hosts of CPXV, caused by spill-over infections from reservoir hosts [18]. Investigations on wild rodents in different European countries identified bank voles (*Myodes glareolus*), field voles (*Microtus agrestis*), common voles (*Microtus arvalis*), striped field mice (*Apodemus agrarius*), wood mice (*Apodemus sylvaticus*), and yellow-necked field mice (*Apodemus flavicollis*) as putative reservoir hosts of CPXV [17,18,19,20,21,22]. They also indicate a seasonality of CPXV infections in rodent populations with peaks in late summer and autumn due to the highest rodent population density at that time [20,21]. Epidemiological investigations of CPXV infection in cats [23,24,25] and zoo animals [17] indicate seasonal peaks during the year, as well, with a major peak in late summer/autumn (August–October) and a smaller one in winter (February–March). Most cats are thought to become infected while hunting wild rodents [23], while elephants, as well as other herbivores are assumed to become infected by feeding on grass, hay, or straw contaminated with rodent feces or urine containing CPXV [26,27].

Thus far, only three cases of CPXV infection of SACs were reported; they described either an individual or small groups of animals being affected [28,29,30].

Between June 2012 and January 2017, four separate clusters of CPXV infections were detected in alpacas from four unrelated herds in Eastern Germany. Common to all, initially one animal with generalized CPXV infection was referred to the Clinic for Ruminants and Swine (Faculty of Veterinary Medicine, University of Leipzig, Leipzig, Germany). Despite intensive care, all four index cases finally succumbed to the disease.

The aim of this study was to investigate the epidemiology of the four clusters of CPXV infection in these alpaca herds. Therefore, the occurrence of CPXV was evaluated in individual alpacas and putative rodent reservoirs by testing CPXV-specific antibodies and DNA and by virus isolation and sequencing. Comparing the results of all four alpaca herds made it possible to draw the first important conclusions on epidemiological aspects like the mode of transmission, the seasonality of the outcome, the role of reservoir hosts, and a possible zoonotic risk.

## 2. Materials and Methods

### 2.1. Alpaca Herds

The study included in total 107 alpacas (37 male; 70 female) of different ages and breeds (104 Huacaya; 3 Suri) out of four herds in Eastern Germany (see Table 1). In three of the four herds, animals are subdivided into different gender-dependent flocks (e.g., stallions, mares, mares and crias), which can vary throughout the year depending on the breeding season. These subdivisions are made by the owners of the herds and do not have any influence on the study. During the time of sampling, there were 8 flocks in herd I, 5 flocks in herd II, and 3 flocks in herd III. There was no further subdivision in herd IV. Direct animal contact between the flocks in each herd was only possible between single flocks (herd I: 2 of 8 flocks, herd II: 2 of 5 flocks, herd III: 2 of 3 flocks). The husbandry systems used were perennial open stabling (herds I, III and IV) and a combination of pasture feeding in summer and open stabling in winter (herd II). Besides pasturage, there was open access to hay and water in all flocks of all four herds. Supplementary feeding of SACs-specific mineral feed or concentrated feed was sporadically done in a herd-dependent manner. Furthermore, other animal species were held in herd III (dogs) and herd IV (cats, dogs, poultry), but not in herds I and II.

### 2.2. Index Patients History

Out of every herd, initially, one animal was referred to the Clinic for Ruminants and Swine, showing symptoms of generalized CPXV infection. These index cases brought the four unrelated clusters of CPXV infection to our attention. Additionally, there was another animal out of herd II referred to the clinic with generalized CPXV infection 65 days after the index case of this herd. All five animals succumbed to the disease. To investigate if and how CPXV spreads throughout the herds, we subsequently visited the corresponding alpaca herds. Each herd was visited twice with clinical examination and sampling of the animals as shown in Table 1.

### 2.3. Sampling

Clinical examination of the alpacas was done according to Baumgartner, 2002, using reference values for SACs as described previously [1,31,32].

Blood samples (serum, ethylenediaminetetraacetic acid (EDTA), lithium-heparin) were obtained by puncture of the jugular vein on the left or right site of the neck, using disposable injection cannulas (B. Braun Melsungen AG, Melsungen, Germany), serum–sample tubes (Sarstedt AG & Co. KG, Nümbrecht, Germany), EDTA–sample tubes, as well as heparin–sample tubes (both, KABE LABORTECHNIK GmbH, Nümbrecht-Elsenroth, Germany). Animals in the clinic were sampled once at the time of their arrival; animals in the herds were sampled as shown in Table 1.

Swab samples were obtained by using dry swabs (KABE LABORTECHNIK GmbH). Depending on the localizations of suspicious pox lesions, swab samples were taken (conjunctiva, nasal mucous membrane, oral mucous membrane, or lesions of the skin, see Table 1).

For virus isolation, crusts of skin lesions were obtained using disposable surgical blades (Bayha GmbH, Tuttigen, Germany) and Cellular Tubes (Greiner Bio-One GmbH, Frickenhausen, Germany). The number of crusts obtained in the herds is shown in Table 1. Furthermore, crusts were obtained from one animal in the clinic (alpaca No. 35, herd II).

After clinical examination, samples of feces were manually obtained out of the rectum of each animal in herds III and IV. These samples were stored in urine specimen cups (KABE LABORTECHNIK GmbH). Swab samples as well as samples of crusts and feces were stored at 4 °C until further processing.

### 2.4. Small Mammals

Trapping permissions were obtained from the concerned lower nature conservation authority for herds I and II (permit number: herd I—AZ 2013-05062-MK, 17, January, 2013; herd II—407.3.3/359.13-22481, 4, April, 2013). Here, small mammals were caught over a period of three consecutive nights using Sherman live traps baited with apple pieces. Traps were placed strategically, covering all paddock borders and landscape singularities that might be attractive to rodents and other small mammals, and were checked twice daily. Trapped small mammals were subsequently anesthetized using carbon dioxide, followed by blood sample extraction and euthanasia. In herds III and IV small mammals were trapped in the house and stable as a private pest control measure using commercially available snap traps baited with apple pieces over a period of three months. Traps were placed, similarly to herds I and II, close to the open stables and on paddock borders, as well as in the house basement of herd III, and were checked once a day. Trapped small mammals were frozen at −80 °C until necropsy. Necropsy followed a standard protocol with the collection of tissue samples in a defined order. Transudates from snap trapped animals were collected during dissection by washing the chest cavity with 1 mL phosphate-buffered saline (PBS). Taxonomic identification was first done morphologically and then verified by cytochrome *b* gene PCR amplification, sequencing, and sequence comparison to GenBank entries as published previously [33]. All tissue samples, sera, and transudates were stored at −20 °C until further processing.

### 2.5. Diagnostic Methods

To detect CPXV-specific antibodies in sera of infected alpacas and in sera/transudates of trapped small mammals, an indirect immunofluorescence assay (iIFA) was performed as previously described [18]. A fluorescein isothiocyanate (FITC)-labeled anti–llama conjugate (Bethyl Laboratories, Inc., Montgomery, TX, USA) was used as a secondary antibody for all alpaca samples. For the sera of four alpacas referred to the clinic (Nos. 35, 37—herd II; No. 216—herd III, No. 306—herd IV) and the sera of infected alpacas of herd III, further dilutions up to 1:32,000 were additionally tested in the iIFA.

Swab samples, feces, crusts, and organ tissues (clinic patients) of the alpacas and tissue samples (liver—herds I + II/nasal septum—herds III + IV) of trapped small mammals were used to perform a quantitative real-time polymerase chain reaction (qPCR) for detecting viral DNA as described before [18,34].

Virus isolation was performed on Vero76 cells (Collection of Cell Lines in Veterinary Medicine (CCLV), Friedrich-Loeffler-Institut, Greifswald-Insel Riems, Germany). Organ material that scored positive in the qPCR was propagated on Vero76 cells. For identification of the OPV species involved, the hemagglutinin (HA) gene was sequenced (CPXV *Ger/2013/Cat/Kira*; CPXV *Ger/2013/Alpaca/DK13/13*; CPXV *Ger/2017/Alpaca/00095_109*) or full-length sequencing of CPXV isolate was conducted as described earlier [12] (CPXV *Ger/2017/common vole FMEimka*). Likewise, the CPXV048 locus (VACV F1L gene homologue) was amplified and sequenced as described before [35]. For phylogenetic analysis, the dataset comprised representative sequences from Old World OPV species camelpox virus, ectromelia virus, monkeypox virus (MPXV), vaccinia virus (VACV), and variola virus (VARV), as well as New World OPV species, i.e., racoonpox virus, skunkpox virus, and volepox virus. Evolutionary analyses were conducted on the basis of the HA gene using MEGA6 software (Molecular Evolutionary Genetics Analysis Version 6.0.) [36] and the complete genomes of the common vole and alpaca isolates (herd III) were compared with blast (basic local alignment search tool) global alignment (https://blast.ncbi.nlm.nih.gov). All generated sequences were deposited under the ENA-Bioproject with Acc.-No. PRJEB23409 (http://www.ebi.ac.uk/ena).

## 3. Results

### 3.1. CPXV Detection in Four Alpaca Herds

#### 3.1.1. Herd I

In alpaca herd I from Thuringia, consisting of 55 alpacas held in eight flocks, the onset of CPXV infection in June 2012 was noticed as one alpaca mare (No. 176) succumbed to generalized CPXV-disease shortly after submission to the Clinic for Ruminants and Swine [29]. The infection was confirmed by a positive CPXV-specific antibody titer of 1:500 and by detection of viral DNA in tissue samples of the lung, the mucosa of the oral cavity, and the mandibular lymph node post mortem [29]. CPXV was isolated (CPXV *Ger/2012/Alpaca Index-Thuringia*), and analysis of the whole genome sequence grouped it into CPXV-like 1 clade of Old World OPVs [12].

Thirty-one days after the index case, 15 of the remaining 54 animals, that had been kept in the same flock as mare No. 176 in June 2012, were also examined (see Table 1 and Table 2). Three of 15 animals (No. 103—flock 4, No. 110—flock 7, No. 165—flock 2) showed CPXV-specific antibodies (at a dilution of 1:500). However, only one of the three animals exhibited focal crusted skin lesions on the face. Unfortunately, these skin lesions were not further investigated for CPXV-specific DNA. Fifty days later in screening the entire herd of 54 animals, five additional alpacas (Nos. 101 and 139—flock 4, Nos. 138 and 145—flock 6, No. 166—flock 7) were tested positive for CPXV-specific antibodies at a dilution of 1:200 and 1:500, respectively. Alpaca No. 103 was again found to be seropositive, now at a dilution of 1:200, whereas alpacas No. 110 and No. 165 were seronegative. Three of six antibody positive animals showed focal crusted skin lesions at that time. These skin lesions were not further investigated for CPXV-specific DNA.

Altogether, there were nine animals (16.4%) out of 55 alpacas in that herd affected by CPXV infection, which belonged to four (flocks 2, 4, 6, and 7) out of eight flocks. Direct animal contact between these infected flocks was not possible, as the flocks were isolated and located around the farmhouse with linear distances of 100 m to ca. 6 km. However, indirect contact through fodder or alpaca-associated utensils cannot be entirely ruled out.

After the second herd investigation, 20 small mammals of five species were trapped (see Table 3): eight striped field mice, one yellow-necked field mouse, six field voles, one bank vole, and four Eurasian pygmy shrews (*Sorex minutus*). Only in five striped field mice were antibodies against CPXV detectable in a dilution of 1:500. None of the small mammals were positive for CPXV-specific DNA.

#### 3.1.2. Herd II

Alpaca herd II in Saxony-Anhalt, consisting of 31 animals held in five flocks, was investigated for CPXV infection after one mare (No. 37), symptomatic of generalized CPXV infection, was referred to the Clinic for Ruminants and Swine (in March 2013) and died. In this mare, infection was confirmed by detection of CPXV-specific antibodies (1:2000) and CPXV-specific DNA in both swab samples of the conjunctiva and lesions of the mucous membrane of the oral cavity. CPXV could be isolated (CPXV *Ger/2013/Alpaca Index-Saxony-Anhalt*) and the whole genome sequence again grouped the isolate into CPXV-like 1 clade of Old World OPVs [12].

One week later, the whole herd of 30 remaining animals was investigated (see Table 1 and Table 2). One alpaca (No. 16—flock 1) tested positive for CPXV-specific antibodies (dilution 1:500). This animal showed focal crusted skin lesions on its nostrils. Unfortunately, these crusts were not further investigated for CPXV-specific DNA, but a swab sample of the oral mucous membrane of this animal was negative for CPXV-specific DNA. Fifty-four days later, two (Nos. 12 and 32—flock 1) out of four animals investigated for some other reason, were found to be seropositive (dilution 1:500). Another two days later, mare No. 35 (flock 1) was referred to the clinic for Ruminants and Swine with a generalized CPXV infection. This animal tested seronegative two months before and was now found to be positive for CPXV-specific antibodies (1:4000). CPXV-specific DNA was detected in both swabs and crusts of skin lesions on the muzzle, and a virus isolate (CPXV *Ger/2013/Alpaca/DK13/13*) was obtained.

In this alpaca herd, there were five animals (16.1%) out of 31 alpacas affected by CPXV infection. These were exclusively found in one of four flocks. Direct animal contact to the other flocks was not possible.

Between the two visits for herd investigations, seven small mammals of two species were trapped (see Table 3): six bank voles, and one Eurasian pygmy shrew. Three bank voles tested positive for CPXV-specific antibodies at dilutions of 1:200 and 1:500, respectively. CPXV-specific DNA was not detected in any of these seven animals.

A cat living in the neighborhood of the alpaca herd was presented to the local veterinarian seven months after the detection of the alpaca index case. This animal showed CPXV-specific clinical signs and CPXV-specific DNA, and a CPXV virus strain was isolated (CPXV *Ger/2013/Cat/Kira*) from a skin sample (See Table 3).

#### 3.1.3. Herd III

In Saxony, an alpaca herd, consisting of 16 animals held in three flocks, came to our attention, because one stallion (No. 216) was referred to the Clinic for Ruminants and Swine with generalized CPXV-infection (in January 2017) and died. The animal did not show antibodies against CPXV. Infection was confirmed by detection of CPXV-specific DNA in tissue samples (mucosa of esophagus and oral cavity) post mortem. CPXV was isolated (CPXV *Ger/2017/Alpaca Index-Saxony*), and analyses of the whole genome sequence also grouped this isolate into CPXV-like 1 clade of Old World OPVs [12].

Seven weeks later, all remaining 15 animals of the herd were examined for the first time (see Table 1 and Table 2). In flock 1, consisting of six mares and one male cria, there was one mare (No. 205) showing focal crusted skin lesions on the muzzle and nostrils. The animal showed CPXV-specific antibodies (1:16,000), and CPXV-specific DNA was detected in swabs and crusts of skin lesions, as well as in a swab of the nasal mucosa and a feces sample. A CPXV strain (CPXV *Ger/2017/Alpaca/00095_109*) was isolated in crusts of the skin lesions. Another mare (No. 201) of the same flock showed sudden onset of acute unilateral muco-purulent kerato-conjunctivitis. CPXV-specific DNA was detected in a conjunctival swab of the infected eye and in a swab of focal alopecia around the nose, but no CPXV-specific antibodies were detected. No CPXV-specific antibodies or DNA were detected in the other five animals of this flock. Flock 2, a group of three stallions, did neither reveal any signs of symptoms of CPXV infection nor of CPXV-specific antibodies or DNA. In flock 3, a group of five stallions (Nos. 211, 212, 213, 214, 215) from which the initial case originated, all animals were found to have high CPXV-specific antibody titers (1:4000–1:32,000), and one stallion (No. 212) tested positive for CPXV-specific DNA in a swab of the nasal mucosa. This stallion had focal crusted skin lesions near the medial canthus of both eyes.

Three weeks later, all 15 alpacas were examined a second time following the same investigation pattern as before. In flock 1, all seven animals (Nos. 201–207) were now found seropositive (dilutions 1:1000–1:16,000), but no further animal with clinical symptoms was recorded. Mare No. 205 was still positive for CPXV-specific DNA in the crusts of the skin lesions. In contrast, CPXV-specific DNA was no longer detectable in the conjunctival swab of mare No. 201. One further animal, the male cria (No. 202), tested positive for CPXV-specific DNA in a conjunctival swab, but did not show any clinical symptoms. For the three stallions in flock 2, still no signs of a CPXV infection were found by either clinical symptoms or CPXV-specific antibodies or DNA. In flock 3, all five stallions stayed seropositive with titers of 1:4000 to 1:32,000. Further molecular investigations revealed crusts of the skin lesions near the medial canthus of both eyes in stallion No. 212 to be positive for CPXV-specific DNA, as well as stallion No. 213 to be positive for CPXV-specific DNA in a single crusted skin lesion on the nose (Table 2).

**Figure 1:**
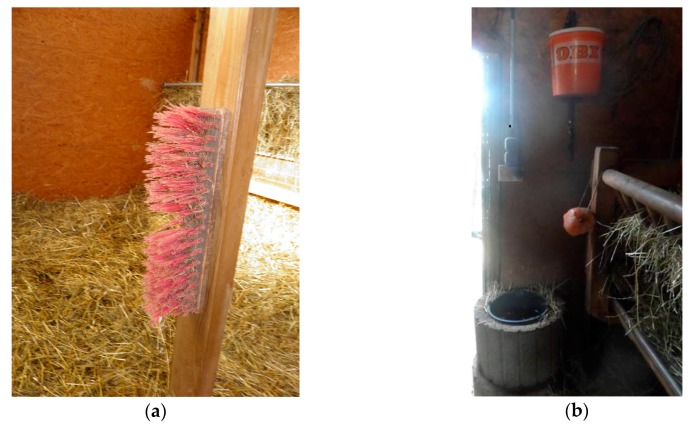
Epidemiological investigations on cowpox virus (CPXV) infection in four alpaca herds in Eastern Germany: (**a**) a broom for grooming, situated in the stable of flock 1 (herd III), tested positive for CPXV-specific DNA by swab sample; (**b**) the water bucket (black) in the stable of flock 3 (herd III), where a dead common vole (*Microtus arvalis*), positive for CPXV-specific DNA, was found.

In total, 13 seropositive animals (81.2%) out of 16 alpacas were found in this herd. All these animals belonged to two of three flocks (flocks 1 and 3). Only the two stallion flocks (flocks 2 and 3) could have direct animal contact through the fence of their paddocks, whereas direct contact to the flock of the mares was not possible, as it was about 100 m away from the other two flocks. Points of intersection between the flocks are the direct contact to the farmer’s family and their clothes, the hay, that is stored on one central stockyard at the farm, and alpaca-associated utensils, like head collars, lead ropes, and scrubbers. There was a broom for grooming, fixed on a wooden beam, situated in the middle of the stable in flock one (see Figure 1a). Swab sampling revealed detection of CPXV-specific DNA on it.

A total of 29 small mammals of six species were trapped at this herd (see Table 3): four striped field mice, two yellow-necked field mice, one bicolored white-toothed shrew (*Crocidura leucodon*), seven common white-toothed shrews (*Crocidura russula*), 14 common voles and one European mole (*Talpa europaea*). In none of these animals were antibodies against CPXV detected. One common vole, however, was positive for CPXV-specific DNA, and a virus was isolated (CPXV *Ger/2017/common vole FMEimka*). This vole was found dead in the water bucket of flock 3 more than two months after a presumably similar event led to the index case of this herd (see Figure 1b). The entire genome sequence of this CPXV strain was determined.

#### 3.1.4. Herd IV

Another case of fatal generalized CPXV infection in an alpaca mare (No. 306) was referred to the Clinic of Ruminants and Swine in January 2017, originating from a small alpaca herd in Brandenburg, with five animals held in one flock. Infection was confirmed by detection of CPXV-specific antibodies (1:500), and post mortem CPXV-specific DNA could be detected in tissue samples of the mucosa of the oral cavity, proventriculus C1, and larynx. Again, a CPXV strain was isolated (CPXV *Ger/2017/Alpaca Index-Brandenburg*) and the whole genome sequence located this isolate into CPXV-like 1 clade of Old World OPVs [12]. Six weeks after the index case, the remaining four animals were investigated for the first time and another four weeks later for the second time (see Table 1). Neither CPXV-specific clinical signs, nor any CPXV-specific antibody or CPXV DNA positive samples were found in any alpaca in both investigations. In the end, there was one infected animal (20%) out of 5 alpacas in this herd, although all 5 animals had direct contact to each other.

Six house mice (*Mus musculus*) were trapped at this alpaca herd (see Table 3). None of these mice was positive for CPXV-specific antibodies or DNA. Out of five cats, living on the farm with supposed close contact to the alpacas, one animal was sampled, but did not show CPXV-specific antibodies or clinical symptoms.

### 3.2. Virus Isolation, Sequence, and Phylogenetic Analyses

**Figure 2:**
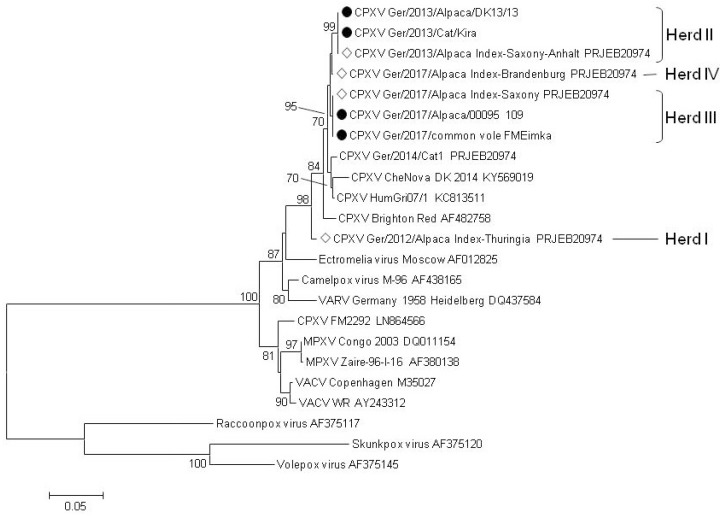
Evolutionary relationship of taxa based on the nucleotide sequence of the hemagglutinin gene. The evolutionary history was inferred using the Neighbor-Joining method [37]. The optimal tree with the sum of branch length = 0.99468564 is shown. The percentage of replicate trees in which the associated taxa clustered together in the bootstrap test (1000 replicates) are shown at the bifurcations [38]. The tree is drawn to scale, with branch lengths in the same units as those of the evolutionary distances used to infer the phylogenetic tree. The evolutionary distances were computed using the Maximum Composite Likelihood method [39] and are in the units of the number of base substitutions per site. The analysis involved 23 nucleotide sequences. All positions containing gaps and missing data were eliminated. There was a total of 629 positions in the final dataset. Evolutionary analyses were conducted in MEGA6 [36]. Abbreviations used are: CPXV, cowpox virus; MPXV, monkeypox virus; VACV, vaccinia virus; VARV, variola virus. Novel sequences generated in this study are indicated by black circles. White diamonds show CPXV isolates from alpacas previously fully sequenced [12].

Skin crust materials previously tested positive for CPXV DNA were used to generate virus isolates originating from alpacas and cat (Table 2 and Table 3). In addition, a common vole isolate was achieved with direct relation to the affected alpaca herd III using material of the vole’s conchae (Table 3). For phylogenetic analysis of the isolated virus strains, the sequences of the HA gene and the complete genomes were generated. Clearly, virus isolates from the local outbreaks cluster together irrespective of the host species (Figure 2). To confirm similarity of the common vole *FM Eimka* origin isolate to the alpaca isolate (CPXV *Ger/2017/Alpaca Index-Saxony*), the whole genome of *FM Eimka* was generated. Interestingly, the similarity value of these isolates from the same location scored 99.997%. CPXV strains isolated from two alpacas of the same herd, but different time points (e.g., CPXV *Ger/2013/Alpaca Index-Saxony-Anhalt* and CPXV *Ger/2013/Alpaca/DK13/13*) exhibited 100% sequence similarity of the HA gene (Figure 2). Phylogenetic clustering was generally supported by high bootstrap values (Figure 2).

In order to further verify the genetic identity of CPXV within each herd, a second locus (CPXV048, which is the VACV F1L gene homologue) was successfully amplified [35], sequenced, and subjected to phylogenetic analyses. Although the overall topology in this tree was slightly different, the clustering of CPXV sequences within each herd was identical as to that of the HA tree (See Figure S1).

## 4. Discussion

In an interval of five years, four separate outbreaks of CPXV infection occurred in four unrelated alpaca herds situated in Eastern Germany. Prior to these, CPXV infection has only been described twice in SACs in Europe [28,30]. These repetitious outbreaks of infection in alpacas needed to be clearly scrutinized by comparing these four clusters and trying to identify epidemiologic markers.

Starting with analysis of the husbandry systems and nutrition, similarities in all four herds were obvious: open stabling with direct open access to pasture except during winter was the preferred method of rearing. The animals were mostly held in flocks with segregation of sexes, and they were fed by grass, hay, and concentrated and mineral feed. Straw was used as litter in the stables. Usually there was one central stockyard for the storage of hay and straw; mineral and concentrated feed is kept in capped buckets or tons.

Considering the seasonality of their outcome, three outbreaks (herd II, III, and IV) occurred in winter and early spring (January till May), whereas one outbreak (herd I) occurred in summer (June till September). Compared to other animal species, this is rather contrary to descriptions in cats [13,23,24,25], where the majority of cases are described in late summer and autumn with a peak in August and September. On the other hand, our findings match the seasonality described for zoo animals [17,40], with CPXV outbreaks occurring in winter (January till March) as well. A likely explanation might be, that rodents (as reservoir hosts) seek immediate proximity to the alpaca stables in winter, because of alimentary and thermal benefits. Thus, the chance for alpacas to come into contact to contaminated fodder by CPXV-infected rodent feces and urine might be higher.

To confirm the involvement of wild rodents as reservoir hosts also in alpaca-associated CPXV outbreaks, trapping of small mammals was successfully performed in all four alpaca herds. In two out of four alpaca herds (herds I and II) a certain number of trapped rodents were found seropositive for CPXV-specific antibodies. Though several species were trapped, in each case, the seropositive rodents belonged to a single species. In Thuringia, they were striped field mice and in Saxony-Anhalt they were bank voles. In accordance to detailed investigations on wild rodents [17,20,21,22,41], these two species are identified as putative reservoir hosts for CPXV in nature. It remains unclear why none of the trapped rodents at the other two herds (Saxony and Brandenburg) were found seropositive for CPXV-specific antibodies. Especially considering that there was one single common vole in herd III, in which the detection of CPXV-specific DNA and the isolation of CPXV was possible, and a comparatively high number of trapped voles there. However, the CPXV positive vole reveals the presence of CPXV in the environment of herd III, as do the seropositive rodents in herds I and II, and the CPXV-infected cat at the neighborhood of herd II. The fact that none of the trapped wild rodents nor the cat in herd IV were seropositive, suggests that CPXV does not circulate extensively in the environment of this herd. Interestingly, all these rodents were house mice, previously not known to be naturally infected with CPXV. Nevertheless, our study provides evidence that voles are putative reservoir hosts of CPXV, and it was again a common vole from which CPXV could be isolated [12]. To the best of our knowledge, common voles are the only natural rodent host species giving rise to CPXV isolates thus far [12,18]. Because of the few CPXV isolates from rodents, it is purely speculative whether common voles shed more (and/or longer) virus than other rodent species, or their life history and preferred habitat is more suitable to the open stabling and pasture feeding commonly used for alpaca herds, or it is simply a coincidence. Rats (*Rattus norwegicus*) are also well known natural hosts for CPXV [11,17], but they were not trapped in our study. The complete genomes of the two CPXV isolates from accidental host (alpaca) and reservoir host (common vole) in herd III were almost 100% identical. This provides further strong evidence for the common vole being the source of the CPXV infection of the alpacas. Here, we assume that the infection route was drinking the water contaminated by the infected vole that was drowned in the water bucket.

Despite the differences that existed between the herds, it should be emphasized that sequences of CPXV were identical within the herds, regardless of whether they originated from an alpaca, a cat, or a vole. This could be demonstrated for herds II and III where three sequences were generated for each. This geographical sequence identity was found for the HA and the F1L homologue gene analyses as well as for the full genome comparison of the vole isolate with the alpaca isolate from the same herd, indicating circulation of a single local CPXV strain. This is further supported by the observation of the owner of herd III who witnessed a drowned rodent in the water bucket shortly before the index case. Sequence identity over the full genome range was almost 100% for the index case and the vole which experienced the same fate more than two months later.

By having a look on the course of infection through the alpaca herds, in all four herds, infection began to appear in one single flock and distributed later among other flocks in two herds. Herd III exhibited more exemplary infection courses and possible ways of transmission. Likely, virus entered the herd by initially infecting one individual through water or fodder contaminated by infectious rodent feces or urine. This theory was nicely affirmed by the CPXV-infected common vole in the water bucket of flock 3. Serology data reveal that after one animal got infected in flock 3, sequentially, all other animals of this flock had seroconverted. The virus was meanwhile also transmitted to the mare flock and one by one the animals seroconverted. As alpacas are so called “distance animals”, avoiding close contact to each other and not performing pair grooming [2,3], direct animal contact as way of virus transmission does not seem obvious (only in case of biting each other, as stallions do while fighting, or in the case of spitting on each other). The fact that none of the stallions of flock 2, which could have direct animal contact to the stallions of flock 3 through the fence, showed positive serology results, supports this. Typical comfort behaviors of SACs are scraping themselves against items in the direct environment (brooms, wood, etc.) and wallowing in the sand [3]. In addition, animals of one flock use the same water bucket and fodder racks; there was a CPXV DNA-positive tested broom in the stable of flock 1, so virus transmission through indirect contact appears to be very likely. As infectious crusts of pox lesions have a high tenacity in the environment [42], indirect transmission, whether by alpaca-associated utensils, by fodder (hay and straw), or maybe also through contact to humans might be possible for a certain time. While these scenarios fit well to the situations in herds I and II, there is no reasonable answer why only one alpaca mare in herd IV got infected and how it became infected.

Interestingly, despite all similarities in the alpaca herds, the dynamic of CPXV infections are diverse. Only one out of five animals in herd IV was infected versus 13 infected out of 16 animals in herd III. This fact raises the question, why does herd III differ from the others? Three herds (herds I, II, and IV) were situated in landscapes that contain mostly plains with fields or pastures and wooden areas close by, only herd III was situated in a valley with the herd property ranging uphill and wooden areas close by. In this herd, rodent holes and burrows were numerous and easy to see on the pastures, and the quantity of trapped rodents was very high. Compared to the other herds, this herd contained the highest quantity of old animals (>10 years of age) emphasizing a possibly higher susceptibility for pathogens in general. Individual health status of the animals is certainly contributing to symptomatic or asymptomatic disease progress [43,44].

CPXV infection as a zoonotic disease always comprises the potential of transmission to humans, especially considering SACs are becoming more and more popular as pets and as animals for animal-assisted therapy or recreational activities like trekking. Although SACs, by their nature, avoid direct contact to other animals as well as to humans, contact with them or with animal-associated utensils while handling or working with them is unavoidable. Our investigations confirm that in a CPXV-infected alpaca, virus is detectable in both various pox lesions (conjunctiva, oral, and nasal mucous membrane, skin) and feces, and that crusts are infectious for at least four weeks. Most of the pox lesions are located on the head, and because of their thick fleece, especially in winter, those can easily be overlooked. As an increasing part of human population is vulnerable for CPXV after termination of cross-protective smallpox vaccination [45], holders of SACs and veterinarians should be aware of CPXV infection as a potential differential diagnosis for symptoms like single, multifocal, or diffuse lesions of the skin or mucous membranes. Cowpox suspicious animals should immediately be separated from the other animals and swab samples and crusts of suspicious pox lesions should be tested. Once CPXV is diagnosed, it is highly recommended to prevent direct human contact (use of disposable gloves) and any animal transfer until the animals test negative for CPXV. Additionally, strict hygiene and disinfection management should be performed among the entire herd before ending quarantine measures. As the husbandry system of alpacas allows continuous contact to wild rodents and voles in particular, a ubiquitous reservoir host of CPXV, virus entry into alpaca herds may be possible at any time, and protective measures, e.g., vaccination of alpacas, might be considered in general.

## Figures and Tables

**Table 1:** Herd specific data and sampling information.

Herd Specific Data	Herd I		Herd II		Herd III		Herd IV	
State	Thuringia		Saxony-Anhalt		Saxony		Brandenburg	
Total number of animals (107)	55		31		16		5	
Gender distribution								
Male (37)	16		9		10		2	
Female (70)	39		22		6		3	
Age distribution								
<1 year (26)	17		6		1		2	
1–10 years (73)	38		23		10		2	
>10 years (8)	0		2		5		1	
Flocks	8		5		3		1	
Husbandry system	Open stabling (perennial)		Pasture feeding (summer), open stabling (winter)		Open stabling (perennial)		Open stabling (perennial)	
Sampling *								
Total number of animals (103) **	54		30		15		4	
Time interval after index Case (in days)	31		9		50		42	
Day of examination (***)	1	2 (50)	1	2 (54)	1	2 (19)	1	2 (28)
Clinical examination	x (15)	x	x	-	x	x	x	x
(Blood) serum	x (15)	x	x	x (4)	x	x	x	x
(Blood) EDTA	x (15)	x	x	-	x	-	x	-
(Blood) lithium-heparin	x (15)	x	x	-	x	-	x	-
Swab (conjunctival)	-	-	-	-	x	x	x	x
Swab (oral mucous membrane)	-	-	x	-	x	x	x	x
Swab (nasal mucous membrane)	-	-	-	-	x	x	x	x
Swab (other)	-	-	-	-	x (8)	x (11)	x (1)	x (3)
Crusts (skin lesions)	-	-	-	-	x (2)	x (3)	x (1)	x (1)
Feces	-	-	-	-	x	x	x	x

* sampling includes two visits per alpaca herd after index case in clinic; ** 4 alpacas (index cases) not included; *** in brackets, time interval (in days) after first examination; x all alpacas of one herd sampled; x ( ) particular number of animals of one herd sampled; - not sampled. EDTA = ethylenediaminetetraacetic acid.

**Table 2:** Clinical signs and results of cowpox virus investigations of infected alpacas from four herds in Eastern Germany.

Herd	Animal Identification **	Day of Examination ***	Sex	Age (Years)	Clinical Signs	iIFA (Highest Dilution Done)	Real-Time PCR/HA Gene Sequence	Virus Isolate
I	176 ^#^	index case	f	5.5	fatal generalization	1:500	+/yes	Ger/2012/Alpaca Index-Thuringia *
	103	1	f	6.2	none	1:500	n.d.	n.d.
		2		6.4	local lesions	1:200	n.d.	n.d.
	110	1	f	4.0	none	1:500	n.d.	n.d.
		2		4.2	local lesions	<1:200	n.d.	n.d.
	165	1	m	0.5	local lesions	1:500	n.d.	n.d.
		2		0.7	local lesions	<1:200	n.d.	n.d.
	101	2	f	3.2	none	1:500	n.d.	n.d.
	138	2	f	5.5	local lesions	1:500	n.d.	n.d.
	139	2	f	9.4	none	1:500	n.d.	n.d.
	145	2	f	7.4	local lesions	1:200	n.d.	n.d.
	166	2	m	0.5	none	1:500	n.d.	n.d.
II	37	index case	f	10.5	fatal generalization	1:2,000	+/yes	Ger/2013/Alpaca Index-Saxony-Anhalt *
	16	1	f	1.6	local lesions	1:500	−	n.d.
	12	2	f	1.8	n.d.	1:500	n.d.	n.d.
	32	2	f	3.9	n.d.	1:500	n.d.	n.d.
	35	clinic	f	10.2	fatal generalization	1:4000	+/yes	Ger/2013/Alpaca/DK13/13
III	216	index case	m	13.1	fatal generalization	<1:200	+/yes	Ger/2017/Alpaca Index-Saxony *
	201	1	f	8.7	unilateral kerato- conjunctivitis	<1:200	+/no	n.d.
		2		8.8	local alopecia	1:16,000	−	n.d.
	205	1	f	12.2	local lesions	1:16,000	+/yes	Ger/2017/Alpaca/ 00095_109
		2		12.2	local lesions	1:16,000	+/no	n.d.
	211	1	m	11.5	none	1:4000	−	n.d.
		2		11.6	none	1:8000	−	n.d.
	212	1	m	8.6	local lesions	1:16,000	+/no	n.d.
		2		8.6	local lesions	1:16,000	+/no	−
	213	1	m	11.8	local alopecia	1:16,000	−	n.d.
		2		11.9	local lesions	1:4000	+/no	−
	214	1	m	7.8	local alopecia	1:4000	−	n.d.
		2		7.9	local alopecia	1:8000	−	n.d.
	215	1	m	8.9	local alopecia	1:32,000	−	n.d.
		2		8.9	local alopecia	1:32,000	−	n.d.
	202	2	m	0.8	none	1:1000	+/no	n.d.
	203	2	f	7.9	local alopecia	1:4000	−	n.d.
	204	2	f	3.6	local alopecia	1:8000	−	n.d.
	206	2	f	8.7	none	1:16,000	−	n.d.
	207	2	f	5.8	local lesions	1:8000	−	n.d.
IV	306	index case	f	10.3	fatal generalization	1:500	+/yes	Ger/2017/Alpaca Index-Brandenburg *

n.d. not done (either real-time PCR was not performed or virus isolation was not attempted when real-time PCR results were negative; clinical examination was not performed); +, positive; −, negative; * virus isolates, that are already described elsewhere [12]; ** animals of each herd are arranged chronologically according to positive results in iIFA or PCR; *** see Table 1 (day of examination); ^#^ published as case report [29]; HA, hemagglutinin; iIFA, indirect immunofluorescence assay; m, male; f, female; Herd I—Thuringia; Herd II—Saxony-Anhalt; Herd III—Saxony; Herd IV—Brandenburg (see Table 1).

**Table 3:** Clinical signs and results of cowpox virus investigations (indirect immunofluorescence assay, real-time PCR and virus isolation) in small mammals and cats from four alpaca herds in Eastern Germany.

Geographic Location	Sample Source	Date	Clinical Signs	Serology (Positive/Tested)		Real-Time PCR/HA Gene Sequence	Virus Isolate
				iIFA 1:200	iIFA 1:500		
Thuringia 2012	*Apodemus agrarius*	20–21 September	none	5/8	5/8	−	n.d.
(Herd I)	*Apodemus flavicollis*	20–21 September	none	0/1	0/1	−	n.d.
	*Microtus agrestis*	20–21 September	none	0/6	0/6	−	n.d.
	*Myodes glareolus*	20 September	none	0/1	0/1	−	n.d.
	*Sorex minutus*	20–21 September	none	0/4	0/4	−	n.d.
Saxony-Anhalt 2013	*Myodes glareolus*	1–2 May	none	3/6	2/6	−	n.d.
(Herd II)	*Sorex minutus*	2 May	none	0/1	0/1	−	n.d.
	Cat	10 October	yes	n.d./1	n.d./1	+/yes	Ger/2013/Cat/Kira
Saxony 2017 (Herd III)	*Apodemus agrarius*	26/27/31 March; 17 July	none	0/4	0/4	−	n.d.
	*Apodemus flavicollis*	20/28 March	none	0/2	0/2	−	n.d.
	*Crocidura leucodon*	19 March	none	0/1	0/1	−	n.d.
	*Crocidura russula*	19–31 March	none	0/7	0/7	−	n.d.
	*Microtus arvalis*	9–26 March; 1 April; 17 July	none	0/11 n.d./3	0/11 n.d./3	+/yes	Ger/2017/common vole FMEimka
	*Talpa europaea*	9 March	none	n.d./1	n.d./1	−	n.d.
Brandenburg 2017	*Mus musculus*	March–July	none	0/6	0/6	−	n.d.
(Herd IV)	Cat	8 March	none	0/1	0/1	n.d.	n.d.

n.d., not done (virus isolation was not attempted when real-time PCR results were negative or real-time PCR was not performed; iIFA was not performed when no transudate or serum of small mammals was available); −, negative; +, positive; HA, hemagglutinin; iIFA, indirect immunofluorescence assay.

## Supplementary Materials

The following are available online at www.mdpi.com/1999-4915/9/11/344/s1.
