# Supplementary material for: Epidemiological Investigations of Four Cowpox Virus Outbreaks in Alpaca Herds, Germany
Source: Viruses. 2017 Nov 18;9(11):344. doi: 10.3390/v9110344 (PMC5707551; doi:10.3390/v9110344)
Supplement: Supplementary file 1 [file viruses-09-00344-s001.pdf]

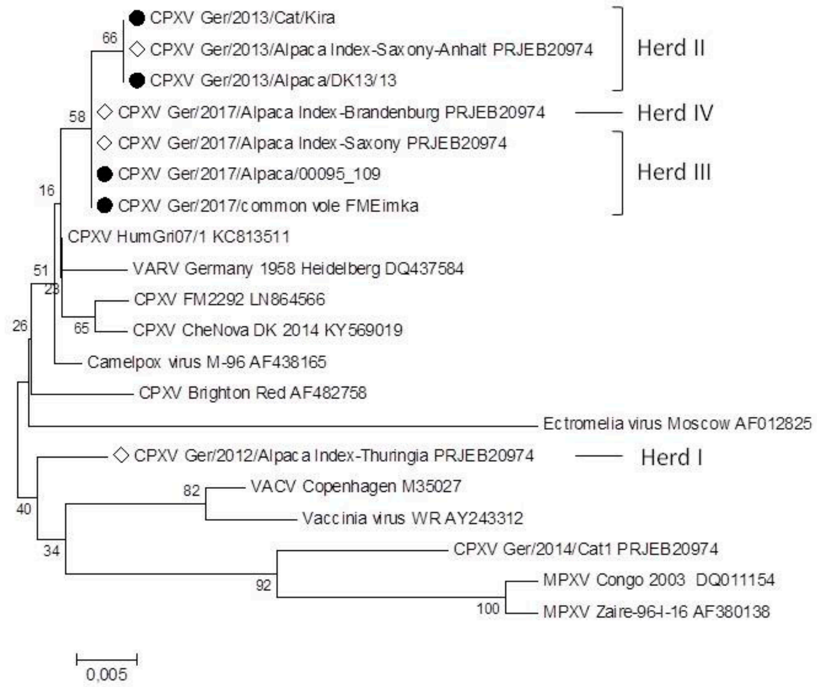

Figure S1. Evolutionary relationship of taxa based on the nucleotide sequence of the CPXV048 (VACV F1L homolog) gene. The evolutionary history was inferred using the Neighbor-Joining method [36]. The optimal tree with the sum of branch length = 0.16460583 is shown. The percentage of replicate trees in which the associated taxa clustered together in the bootstrap test (1000 replicates) is shown at the bifurcations [44]. The tree is drawn to scale, with branch lengths in the same units as those of the evolutionary distances used to infer the phylogenetic tree. The evolutionary distances were computed using the Maximum Composite Likelihood method [37] and are in the units of the number of base substitutions per site. The analysis involved 20 nucleotide sequences. Codon positions included were 1st+2nd+3rd+noncoding. All positions containing gaps and missing data were eliminated. There was a total of 371 positions in the final dataset. Evolutionary analyses were conducted in MEGA6 [35]. Abbreviations used are: CPXV, cowpox virus; MPXV, monkeypox virus; VACV, vaccinia virus; VARV, variola virus. Novel sequences generated in this study are indicated by black circles. White diamonds show CPXV isolates from alpacas previously fully sequenced [12].
